# Characterization of Flow Behaviors by a PSO-BP Integrated Model for a Medium Carbon Alloy Steel

**DOI:** 10.3390/ma16082982

**Published:** 2023-04-09

**Authors:** Guozheng Quan, Yu Zhang, Sheng Lei, Wei Xiong

**Affiliations:** 1 Chongqing Key Laboratory of Advanced Mold Intelligent Manufacturing, School of Material Science and Engineering, Chongqing University, Chongqing 400044, China; 2 Key Laboratory of Advanced Reactor Engineering and Safety of Ministry of Education, Collaborative Innovation Center of Advanced Nuclear Energy Technology, Institute of Nuclear and New Energy Technology, Tsinghua University, Beijing 100084, China

**Keywords:** SAE 5137H steel, flow stress, Arrhenius-type equation, BP-ANN, PSO-BP

## Abstract

In order to characterize the flow behaviors of SAE 5137H steel, isothermal compression tests at the temperatures of 1123 K, 1213 K, 1303 K, 1393 K, and 1483 K, and the strain rates of 0.01 s^−1^, 0.1 s^−1^, 1 s^−1^, and 10 s^−1^ were performed using a Gleeble 3500 thermo-mechanical simulator. The analysis results of true stress-strain curves show that the flow stress decreases with temperature increasing and strain rate decreasing. In order to accurately and efficiently characterize the complex flow behaviors, the intelligent learning method backpropagation–artificial neural network (BP-ANN) was combined with the particle swarm optimization (PSO), namely, the PSO-BP integrated model. Detailed comparisons of the semi-physical model with improved Arrhenius-Type, BP-ANN, and PSO-BP integrated model for the flow behaviors of SAE 5137H steel in terms of generative ability, predictive ability, and modeling efficiency were presented. The comparison results show that the PSO-BP integrated model has the best comprehensive ability, BP-ANN is the second, and semi-physical model with improved Arrhenius-Type is the lowest. It indicates that the PSO-BP integrated model can accurately describe the flow behaviors of SAE 5137H steel.

## 1. Introduction

SAE 5137H is a medium carbon alloy steel with high strength that is often used in the manufacture of industrial gears. It is one of the key materials with high requirements for the core components to ensure safety such as gear, knuckle, etc. in the fields of automobiles, railways, ships and construction machinery [1,2,3]. These core components need to be formed by hot forging to obtain excellent service performance. In order to enclose a perfect design for this process, the accurate numerical computation is always pursued to acquire the physical field distribution and its evolution [4]. Consequently, it is significant for the finite element modeling of the forming processes to deeply understand and accurately characterize the complex flow stress behaviors in a wide range of deformation conditions including temperature, strain rate, and strain. It is commonly considered as a basic work.

A large number of studies have shown that physical models are widely used to describe the complex flow behaviors of metals, such as Johnson-Cook type [5,6], Arrhenius type [7,8], Bergstrom type [9], Kolmogorov–Johnson–Mehl–Avrami type [10], etc. The physical model is based on a set of parameters that can be expressed as a function of forming temperature, strain, and strain rate. These functions consider the effect of these forming parameters on the flow behaviors of a metal. The physical models have theoretical foundations, for example, sufficient understanding of the physical mechanisms and logical relationships between parameters. Lin et al. [11] developed a physically constitutive model considering dislocation density, and Voyiadjis et al. [12] developed a microstructure-based physically constitutive model considering the effect of mobile dislocation density evolution on the flow stress. Atef et al. [13] and Mohammed et al. [14] used the dislocation density-based Bergstrom and the diffusional transformation-based Kolmogorov-Johnson-Mehl-Avrami models to characterize the hot deformation behavior of the steels. These studies show that if the complex microscopic deformation mechanisms were ignored, the predicted stress values would deviate from the actual ones.

Later, semi-physical models that are partially based on phenomenology were gradually developed. The semi-physical models are a family of models that are built based on the knowledge of the described phenomena. Semi-physical models do not require in-depth knowledge of complex microscopic deformation mechanisms. It is only required to calculate the necessary material constants from limited experimental data to construct multiple nonlinear regression models. Some of the calculated material constants have no practical physical significance. Zhao et al. [15] proposed an improved constitutive relationship to characterize the rheological behaviors at low and medium temperature ranges with variable strain rates based on the Johnson-Cook model. Lin et al. [16] considered the effect of different strains on stresses, and then introduced a series of variable coefficients that change with strain (including activation energy of deformation *Q*, material constants *n* and *α*, and structure factor *A*) into Arrhenius equation through polynomial fitting. In this model, *Q* and *A* are the physical parameters, while *n* and *α* are the phenomenological parameters. Semi-physical models have lower prediction accuracy in predicting unknown deformation conditions.

In recent years, intelligent models with simple and efficient modeling were widely used in flow behaviors models. The widely used model backpropagation–artificial neural network (BP-ANN) is an intelligent algorithm for biological neural systems [17,18,19]. BP-ANN can achieve high accuracy levels, but a large number of network topologies and training parameters need to be tried to obtain higher accuracy, which will consume a lot of time and effort. In addition, neural networks are not always stable. For a certain dataset, the accuracy obtained with different attempts of the same network topology and neural network training parameters fluctuates. BP-ANN has a large global range in the process of optimally exploring the initial values of weights and thresholds, which reduces the modeling efficiency. In addition, BP-ANN is prone to fall into local extremes and cannot obtain global optimal solutions.

In order to overcome the shortcomings of a single algorithm, the use of multiple algorithm fusion is the current general trend [20,21]. For example, particle swarm optimization (PSO) was introduced into BP-ANN to form a PSO-BP integrated model. The principle of the PSO-BP integrated model is to first search for the particle-optimal solution by PSO, and then use the particle-optimal solution outputs as the initial thresholds and weights of BP-ANN for training, and finally the optimal solutions are obtained [22,23]. The PSO-BP integrated model can overcome the disadvantage that a single BP-ANN can easily fall into local extremes, and can help the BP-ANN find the optimal solution more quickly. Therefore, the PSO-BP integrated model greatly improves modeling efficiency, modeling accuracy and modeling stability.

In this work, the generative abilities, predictive abilities, and modeling efficiencies among the improved Arrhenius-type constitutive model, BP-ANN, and PSO-BP integrated model were evaluated using correlation coefficients (*R*), relative error (δ), standard deviation (w) and so on. The results show that both BP-ANN and PSO-BP integrated model were able to learn the flow behaviors with sufficient accuracy and provide accurate prediction results. The PSO algorithm quickly and accurately obtains the weights and thresholds of the BP-ANN and improves efficiency and accuracy; therefore, the PSO-BP integrated model has superior comprehensive abilities. Compared to them, the semi-physical model with improved Arrhenius-Type has higher errors and cannot accurately predict the flow behaviors of SAE 5137H steel. In finite element software, if the software needs to invoke stress-strain data, the stress-strain data required but not entered are calculated mainly by interpolation methods. However, the prediction results of this method have large errors. The prediction of stresses outside experimental conditions by the PSO-BP integrated model can improve related research areas where stress-strain data play an important role.

## 2. Proposed PSO-BP Integrated Model and Principles

### 2.1. The Basic Principles of BP-ANN

**Figure 1:**
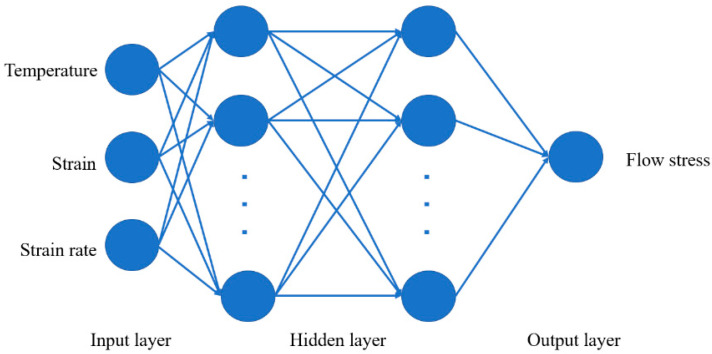
The basic structure of the BP-ANN.

Artificial neural network (ANN) is an error feedback neural network algorithm based on the model of a human neuron [24]. The human brain consists of millions of neurons which sends and processes signals in the form of electrical and chemical signals. These neurons are connected with a special structure known as synapses. Synapses allow neurons to pass signals from large numbers of simulated neurons in neural networks forms. Backpropagation–artificial neural network (BP-ANN) is a kind of ANN that can be divided into two processes, forward propagation and backward propagation, using gradient descent to achieve the adjustment of different neural layer weights to achieve the effect of input feature vectors and output categories. In terms of structure, BP-ANN is composed of two modules: a forward propagation network and an error back propagation network. The basic structure of BP-ANN is shown in Figure 1. BP-ANN consists of three main layers, including an input layer, a hidden layer and an output layer. Various information from the outside is transmitted through the input layer of the BP-ANN to its hidden layer. The hidden layer receives the data and operates according to the user-selected transfer function, and then transmits the data to the next layer, and so on. The next layer is similarly passed layer by layer to the output layer, and finally the result of the operation is obtained, which is the generation of a training. When the error between the output result of the output layer of the BP-ANN and its preset input value is large, it enters the back-propagation phase of the BP-ANN and updates the network weights until the error between the output result and the expected result satisfies certain conditions. BP-ANN is used to establish the corresponding constitutive model through the mapping relationship between deformation parameters and stresses, without giving the model in advance. It can find the law directly from a large amount of data, and automatically adjust the weights and thresholds in the network through training to match the network model adapted to the experimental data.

### 2.2. The Basic Principles of PSO-BP Integrated Model

Particle swarm optimization (PSO) is a kind of evolutionary algorithm developed by imitating the foraging behavior of flocks of birds and fish [25]. Its concept is simple and easy to program and implement with high operational efficiency and relatively few parameters, and it is very widely used. The position of each particle in the PSO represents a candidate solution to the problem to be solved. The PSO has the ability for each particle to learn itself and for particles to collectively learn from each other in real time. At the same time, the particles are constantly sharing information among themselves in real time and have the ability to communicate with each other and the environment, thus changing their own and collective behavior. The position information of particles is usually determined by two factors, one is the information of particle motion speed and the other is the information of relative position of particles. Each particle realizes real-time information sharing based on mutual learning in the whole particle swarm, and then constantly changes the position information of individual particles.

Since the BP-ANN training uses the gradient-down method for parameter iteration, it is inevitable that the problem of slow convergence and easy to fall into local optimal solutions will occur. In this paper, the PSO is introduced into the BP-ANN. The parameters to be adjusted in the BP-ANN are used as the particle positions in the PSO, and the mean square error values of the predicted and actual values from the training samples are used as the fitness functions of the PSO algorithm. After several iterations, the optimal particle positions are found to obtain the optimal BP-ANN structure, which improves the model detection accuracy.

**Figure 2:**
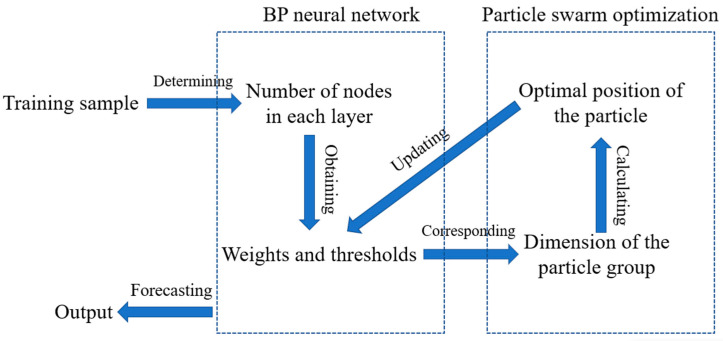
Principle of the PSO-BP integrated model.

Figure 2 shows the principle of combining the BP-ANN and PSO, it can be summarized as follows. First, a global search PSO is used for assistance to accelerate the global exploration phase, which can solve the problem that the BP-ANN tends to fall into local optimal solutions. After the PSO processing, the location of the optimal solution with initial values of weights and thresholds will be in a relatively small search range. Then, the traditional BP-ANN training phase is performed again to explore the local optimal solutions in the current range, using the particle optimal solutions output by the PSO as the initial threshold and weights. After continuously adjusting the parameters, the weights and thresholds of the neural network model are modified until the end condition is satisfied. The PSO-BP integrated model training is finished.

## 3. Modeling the Constitutive Model for SAE 5137H Steel

### 3.1. Acquisition of Experimental Stress-Strain Data

**Figure 3:**
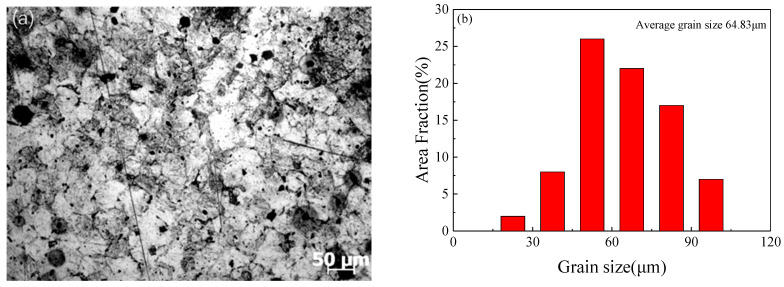
(**a**) Initial microstructure and (**b**) grain size distribution of the SAE 5137H steel.

**Figure 4:**
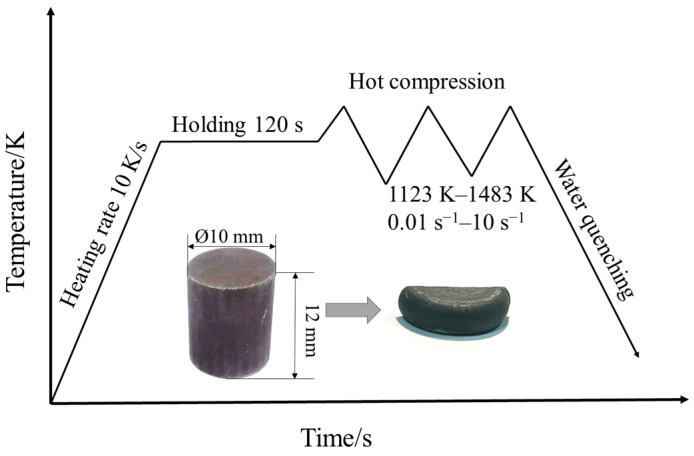
Isothermal compression deformation process.

The specific chemical composition (wt.%) of SAE 5137H steel, as provided through the manufacturer, was listed in Table 1. The initial microstructure with an average grain size of 64.8 µm was exhibited in Figure 3. The samples were cylinders from rolled billet with a diameter of 10 mm and a height of 12 mm. The stress-strain data of this steel was acquired from a type of isothermal compression test at a certain temperature and a certain strain rate. A computer-controlled servo-hydraulic thermos-mechanical machine, i.e., Gleeble 3500, with a stable heating system, was adopted for this isothermal compression. The experiment procedures were roughly shown in Figure 4. First of all, tantalum sheets were padded at both ends of the specimen before the experiment to minimize the friction between the edges of the specimen and the dies. Then, a specimen was heated to the proposal temperature at a rate of 10 K/s and held at a fixed temperature for 3 min. The holding treatment was to ensure that specimen achieved uniform temperature at the onset of a compression process, which could reduce the anisotropy of the material in flow deformation behavior. In addition, two thermocouple wires were welded to two points in the middle of each specimen before the test and the two wires will feedback the changes of temperature of the specimen at any time to the control system of Gleeble 3500, which is convenient for the system to adjust Itself and accurately control the temperature during text. Referring to the actual range of forging process parameters used in the factory, the temperatures of the compression test were selected as 1123 K, 1213 K, 1303 K, 1393 K, and 1483 K, and the strain rates were selected as 0.01 s^−1^, 0.1 s^−1^, 1 s^−1^, and 10 s^−1^. According to the material properties of SAE 5137H, the compression ratio of height should reach 60% during the test. Finally, the deformed specimens were immediately quenched with water to room temperature to preserve the elevated temperature microstructures. More details related to the isothermal compression procedures can be obtained in references [26].

**Figure 5:**
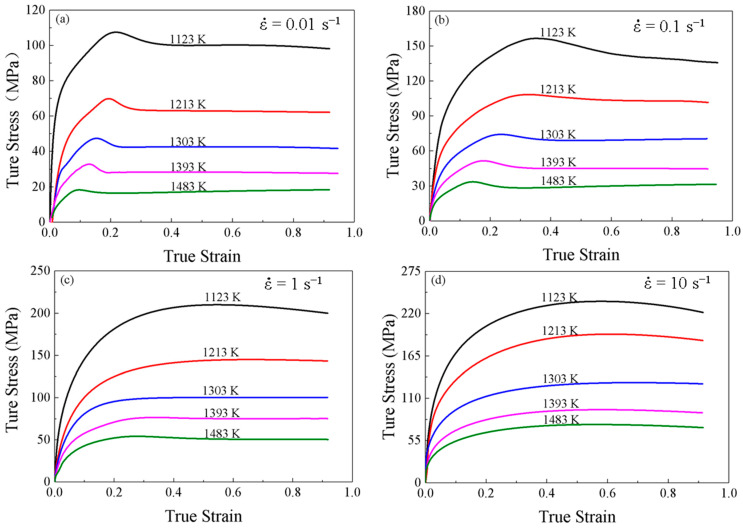
True strain-stress curves for SAE 5137H steel under different deformation temperatures and strain rates of (**a**) 0.01 s^−1^, (**b**) 0.1 s^−1^, (**c**) 1 s^−1^ and (**d**) 10 s^−1^.

The true stress-strain curves of SAE 5137H steel during the compression processes under different deformation conditions are shown as Figure 5. Considering adiabatic heating during the deformation at high strain rates may significantly influence the true stress–true strain curves, the reliability of the compression experimental results was judged using the expansion coefficient in Equation (1). When the expansion coefficient *B* > 0.9 is considered reliable. On the contrary, it is not reliable, and the stress value obtained is corrected with Equation (2). After the test, expansion coefficient of all specimens after this isothermal compression test were greater than 0.9. Therefore, the results obtained from this experiment were reliable and can be used directly.

(1) $$B_{0}=\frac{L_{0}d_{0}^{2}}{L_{f}d_{f}^{2}}$$

where *B* is expansion coefficient, *L*_0_ and *L_f_* are the original height of the specimen and the height after compression deformation, respectively, d_0_ and d*_f_* are the original diameter of the specimen and the diameter after compression deformation, respectively.

(2) $$\sigma_{i}=\frac{4F_{i}}{\pi d_{i}^{2}}\left(1+\frac{\mu d_{i}}{3L_{i}}\right)$$

where *σ_i_* is corrected true stress, *F_i_*, *di*, *L_i_*, and μ are the pressure on the specimen, the average diameter, the average height, and the friction coefficient, respectively.

The variation in flow stress with true strain can be summarized in three stages. In the first stage, the flow stress increases rapidly to a critical value with the true strain increases, and work hardening (WH) is the predominant deformation mechanism. At the same time, the grain boundary storage energy increases rapidly to the dynamic recrystallization activation energy (DRX). In the second stage, DRX and dynamic recovery (DRV) occur and increase, and the rate of increase of flow stress decreases until the maximum stress is reached. At this point, thermal softening begins to exceed WH. In the last stage, two types of stress change patterns are observed. Flow stress continues to decrease and the DRX softens obviously at strain rates of 0.01 and 0.1. Different from the first type, true stress-strain curves at strain rates of 1 and 10 do not show significant stress peaks. With the increase of true strain, the true stress basically remains stable due to the dynamic equilibrium of the softening behaviors of WH and DRV. At higher temperatures and lower strain rates, the flow stress is relatively lower due to sufficient time for recrystallization gain for energy accumulation and nucleation. In addition, high temperature accelerates dislocation movement and grain boundary migration. The flow behaviors are nonlinear with complex deformation mechanism. Therefore, it is important to establish a constitutive model to characterize the flow behaviors of SAE 5137H steel.

### 3.2. Semi-Physical Model with Improved Arrhenius-Type for Flow Behavior of SAE 5137H Steel

As for the Arrhenius-type constitutive equation, the effects of the temperatures and strain rates on the deformation behaviors are represented by Zener-Hollomon parameter, *Z*, in an exponent-type expression.

(3) $$Z=\left|\dot{\varepsilon }\right|\exp\left[Q/\left(RT\right)\right]$$

where $\dot{\varepsilon }$ is strain rate (s^−1^). *Q* is the activation energy of hot deformation (kJ·mol^−1^), *A* is material constant, *R* is the universal gas constant (8.31 J·mol^−1^·K^−1^), *T* is the absolute temperature (K), $F\left(\sigma \right)$ is the function of stress, it takes three forms, and it is expressed as Equation (4),

(4) $$F\left(\sigma \right)\left\{\begin{array}{ll} {\left|\sigma \right|}^{n} & \alpha \left|\sigma \right|<0.8\\ \exp\left(\beta \left|\sigma \right|\right) & \alpha \left|\sigma \right|<0.8\\ \left[\sinh\left(\alpha \left|\sigma \right|\right)\right] & for\;all\;\left|\sigma \right| \end{array}\right.$$

where α, β, and n are the material constants, α=β/n. σ is the flow stress (MPa) for a given strain.

For the low stress level $\left(\alpha \left|\sigma \right|<0.8\right)$, substituting ${\left|\sigma \right|}^{n}$ into Equation (3), respectively, gives (5). Similarly for the high stress level $\left(\alpha \left|\sigma \right|>1.2\right)$, substituting $\exp\left(\beta \left|\sigma \right|\right)$ into Equation (3), gives Equation (6).

(5) $$\left|\dot{\varepsilon }\right|=A{\left|\sigma \right|}^{n}\exp\left[-Q/\left(RT\right)\right]$$

(6) $$\left|\dot{\varepsilon }\right|=A\exp\left(\beta \left|\sigma \right|\right)\exp\left[-Q/\left(RT\right)\right]$$

Taking the logarithm on both sides of Equations (5) and (6), we have,

(7) $$\ln\left|\sigma \right|=\frac{1}{n}\ln\left|\dot{\varepsilon }\right|+\frac{1}{n}\left(\frac{Q}{RT}-\ln A\right)$$

(8) $$\left|\sigma \right|=\frac{1}{\beta }\ln\left|\dot{\varepsilon }\right|+\frac{1}{\beta }\left(\frac{Q}{RT}-\ln A\right)$$

**Figure 6:**
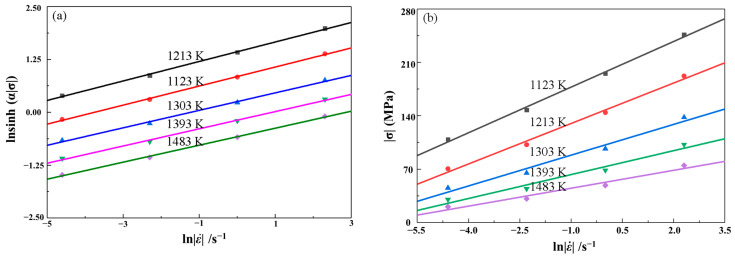
Relationships between strain rates and peak stresses: (**a**) $\ln\left|\dot{\varepsilon }\right|$ and $\ln\left|\sigma \right|$; (**b**) $\ln\left|\dot{\varepsilon }\right|$ and $\left|\sigma \right|$.

Consequently, the slope of $\ln\left|\sigma \right|$ versus $\ln\left|\dot{\varepsilon }\right|$ and $\left|\sigma \right|$ versus $\ln\left|\dot{\varepsilon }\right|$ gives the value of n and β. This means $n=d\ln\left|\dot{\varepsilon }\right|/d\ln\left|\sigma \right|$, $\beta =d\ln\left|\dot{\varepsilon }\right|/\left|\sigma \right|$, respectively. Then, substituting the values of the peak stress and corresponding strain rates into the logarithm Equation (7) gives the relationships of $\ln\left|\dot{\varepsilon }\right|-\ln\left|\sigma \right|$ as shown in Figure 6a. It is not difficult to find that the natural logarithms of stresses at every temperature are linear and the slopes are approximated the same with each other. The average value of all the lines’ slopes can be regarded as the inverse of n, thus $n={6.0083\;\mathrm{MPa}}^{-1}$. Meanwhile, substituting the values of the peak stress and corresponding strain rates into the logarithm Equation (8) gives the relationships of $\ln\left|\dot{\varepsilon }\right|-\left|\sigma \right|$ as shown in Figure 6b. For the same, stresses at every temperature are linear and the average value of all the lines’ slopes can be regarded as the inverse of β, thus $\beta ={0.0655\;\mathrm{MPa}}^{-1}$. Then, α=β/n=0.0109.

Substituting the hyperbolic law of $F\left(\sigma \right)$ into Equation (2) gives,

(9) $$\left|\dot{\varepsilon }\right|=A{\left[\sinh\left(\alpha \left|\sigma \right|\right)\right]}^{n}\exp\left[-Q/\left(RT\right)\right]$$

Taking the natural logarithm of both sides of Equation (7) gives,

(10) $$\ln\left|\dot{\varepsilon }\right|=\ln A+n\sinh\left(\alpha \left|\sigma \right|\right)-Q/\left(RT\right)$$

By linear fit, Equation (10) can be rewritten as,

(11) $$Q=Rn\left\{d\left[\ln\sinh\alpha \left|\sigma \right|\right]/d\left(1/T\right)\right\}$$

**Figure 7:**
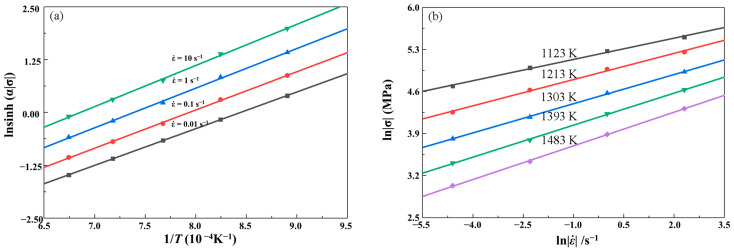
Relationships between: (**a**) $\ln\sinh\left[\alpha \left|\sigma \right|\right]$ and 1/*T*; (**b**) $\ln\sinh\left[\alpha \left|\sigma \right|\right]$ and $\ln\left|\dot{\varepsilon }\right|$.

As shown in Figure 7a, the distribution of all points is linear. By a linear fitting with an average error of 0.10, the relationships between $\ln\sinh\left[\alpha \left|\sigma \right|\right]$ and 1/*T* is linear at different strain rate, and the slopes are approximated the same with each other. The value of *Q* can be obtained from the slope of $\ln\sinh\alpha \left|\sigma \right|$ versus 1/*T.* The average value of all the slope rates is accepted *Q*/(*RT*), furthermore, the value of *Q* is obtained as 359.6095 kJ·mol^−1^.

Equation (10) can also be expressed as following:

(12) $$\ln\sinh\left(\alpha \left|\sigma \right|\right)=\frac{1}{n}\ln\left|\dot{\varepsilon }\right|+\frac{1}{n}\left(\frac{Q}{RT}-\ln A\right)$$

By substituting the values of the peak stress at different temperatures and strain rates into Equation (12), the linear relationships between $\ln\sinh\left[\alpha \left|\sigma \right|\right]$ and $\ln\dot{\varepsilon }$ for different temperatures can be obtained as shown in Figure 7b. The average value of all the intercepts of $\ln\sinh\alpha \left|\sigma \right|$ versus $\ln\dot{\varepsilon }$ plot is obtained as the value of *A*, thus *A* value is calculated as 6.9633 × 10^13^ s^−1^. Submitting the values of material constants α, n, *Q*, and *A* into Equation (9) gives,

(13) $$\left|\dot{\varepsilon }\right|=6.9633\times 10^{13}{\left[\sinh\left(0.0109\left|\sigma \right|\right)\right]}^{6.0083}\exp\left[-\left(359.6095\times 10^{13}\right)/RT\right]$$

Substituting Equation (3) into Equation (4), then the flow stress can be expressed as Equation (14).

(14) $$\left|\sigma \right|=\frac{1}{\alpha }\ln\left\{{\left(\frac{Z}{A}\right)}^{\frac{1}{n}}+{\left[{\left(\frac{Z}{A}\right)}^{\frac{2}{n}}+1\right]}^{\frac{1}{2}}\right\}$$

Substituting Equation (3) and *A* into Equation (14), the constitutive equation of flow stress for SAE 5137H steel can be calculated as Equation (15).

(15) $$\left|\sigma \right|=91.7431\ln\left\{{\left(\frac{\left|\dot{\varepsilon }\right|\exp\left[\frac{359.6095\times 10^{3}}{RT}\right]}{6.9633\times 10^{13}}\right)}^{\frac{1}{6.0083}}+{\left[{\left(\frac{\left|\dot{\varepsilon }\right|\exp\left[\frac{359.6095\times 10^{3}}{RT}\right]}{6.9633\times 10^{13}}\right)}^{\frac{2}{6.0083}}+1\right]}^{\frac{1}{2}}\right\}$$

(16) $$\left\{\begin{array}{l} Q\left(\varepsilon \right)=B_{0}+B_{1}\varepsilon +B_{2}\varepsilon^{2}+B_{3}\varepsilon^{3}+B_{4}\varepsilon^{4}+B_{5}\varepsilon^{5}+B_{6}\varepsilon^{6}+B_{7}\varepsilon^{7}\\ n\left(\varepsilon \right)=C_{0}+C_{1}\varepsilon +C_{2}\varepsilon^{2}+C_{3}\varepsilon^{3}+C_{4}\varepsilon^{4}+C_{5}\varepsilon^{5}+C_{6}\varepsilon^{6}+C_{7}\varepsilon^{7}\\ \ln A\left(\varepsilon \right)=D_{0}+D_{1}\varepsilon +D_{2}\varepsilon^{2}+D_{3}\varepsilon^{3}+D_{4}\varepsilon^{4}+D_{5}\varepsilon^{5}+D_{6}\varepsilon^{6}+D_{7}\varepsilon^{7}\\ \alpha \left(\varepsilon \right)=E_{0}+E_{1}\varepsilon +E_{2}\varepsilon^{2}+E_{3}\varepsilon^{3}+E_{4}\varepsilon^{4}+E_{5}\varepsilon^{5}+E_{6}\varepsilon^{6}+E_{7}\varepsilon^{7} \end{array}\right.$$

Substituting the polynomial functions of $Q\left(\varepsilon \right)$, $n\left(\varepsilon \right)$, $A\left(\varepsilon \right)$ and $\alpha \left(\varepsilon \right)$ into Equation (9), and gives Equation (17).

(17) $$\left|\dot{\varepsilon }\right|=A\left(\varepsilon \right){\left[\sinh\left(\alpha \left(\varepsilon \right)\left|\sigma \right|\right)\right]}^{n\left(\varepsilon \right)}\exp\left[-Q\left(\varepsilon \right)/\left(RT\right)\right]$$

Finally, the Arrhenius type equation of SAE 5137H steel can be developed as following:

(18) $$\left|\sigma \right|=\frac{1}{\alpha \left(\varepsilon \right)}\ln\left\{{\left(\frac{\left|\dot{\varepsilon }\right|\exp\left[Q\left(\varepsilon \right)/RT\right]}{A\left(\varepsilon \right)}\right)}^{\frac{1}{n\left(\varepsilon \right)}}+{\left[{\left(\frac{\left|\dot{\varepsilon }\right|\exp\left[Q\left(\varepsilon \right)/RT\right]}{A\left(\varepsilon \right)}\right)}^{\frac{2}{n\left(\varepsilon \right)}}+1\right]}^{\frac{1}{2}}\right\}$$

**Figure 8:**
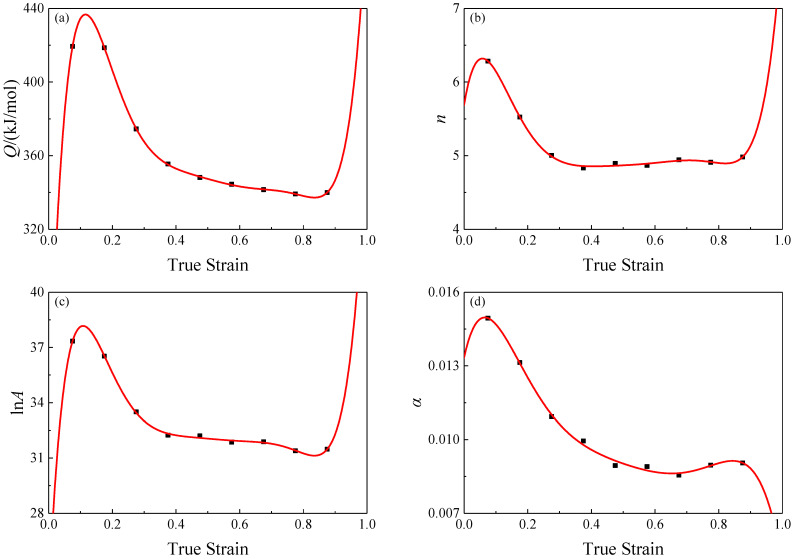
Relationships between: (**a**) $Q\left(\varepsilon \right)$; (**b**) $n\left(\varepsilon \right)$; (**c**) $\ln A\left(\varepsilon \right)$; (**d**) $\alpha \left(\varepsilon \right)$ and true strain by polynomial fitted of SAE 5137H steel.

The above is the calculation process of the Arrhenius-type constitutive equation for SAE 5137H steel, while this equation ignores the effect of strain on the flow stress, and then this equation is lack of the ability to predict the stresses at different strains. In order to solve this issue, the strain compensation is introduced by constructing a series of polynomials as Equation (16) representing the nonlinear relationships between the variables (including activation energy of deformation *Q*, material constants *n*, and *α*, and structure factor *A*) in Arrhenius-type constitutive equation and strains. In order to find the variation pattern of the variables, the values of the variables were fitted nonlinearly at 0.1 true strain interval. Such nonlinear relationships were shown as Figure 8, and the coefficients of the fitted polynomials were listed in Table 2.

### 3.3. BP-ANN for Flow Behavior of SAE 5137H Steel

In this investigation, the BP-ANN was developed by MATLAB software. The input variables include deformation temperatures and strains, and the output variables were flow stresses. The 20 curves were divided into two datasets, i.e., the training dataset and the test dataset, as shown in Table 3. A total of 308 input-output pairs were selected from the stress-strain curves to train and test the BP-ANN. The 36 stress points on the test stress-strain curves in the strain range 0.075~0.875 with a distance of 0.1 were not used for training, but for testing the BP-ANN generation ability. The BP-ANN was trained using 272 stress points in the strain range of 0.05~0.85 and distance of 0.5 in the training stress-strain curves. Due to the different units of measurement from experimental data such as temperatures, strains, strain rates, and stresses, there were large differences between different types of data, and such differences would reduce the speed and accuracy of convergence within the network. Therefore, the input and output datasets measured in different units need to be normalized to dimensionless units before the networks are trained to eliminate the arbitrary effect of similarity between different data. The input and output data were normalized in the range of 0~1 according to the relationship given in Equation (19).

(19) $$y_{n}=\frac{y-0.95y_{\min}}{1.25y_{\max}-0.95y_{\min}}$$

where $y_{n}$ is the normalized value of y, y is the experimental data, $y_{\max}$ and $y_{\min}$ are the maximum and minimum value of y respectively.

As mentioned above, for a typical BP-ANN structure, one or more hidden layers were required, and two hidden layers were used here to ensure a high training accuracy. In addition, the number of nodes in the input layer was 3, the number of nodes in the hidden layer was 12, and the number of nodes in the output layer was 1. Considering the range of values, the tansig was used as hidden layers function, and the purelin was used as the output layer function. Other parameters were shown in Table 4.

### 3.4. PSO-BP Integrated Model for Flow Behavior of SAE 5137H Steel

**Figure 9:**
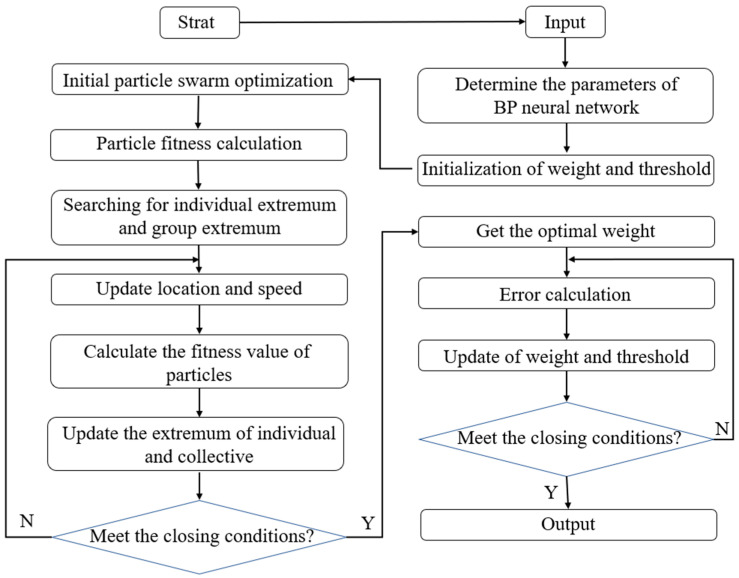
Flow of the PSO-BP integrated model algorithm.

Figure 9 describes the overall processes of the PSO-BP integrated model. First, the parameters of the algorithm are determined. Based on the input and output datasets of the stress-strain curves, the topology of the BP-ANN and the initial values of the PSO algorithm are determined. The connection weights and closure values between all neurons are encoded as vectors of real numbers to represent the individual particles in the population. In the second, the mean square error of the training output of the BP-ANN with respect to the sample output is calculated according to the fitness function. The optimal value of each particle is updated. The velocity of each particle is adjusted according to the velocity update formula, and the position of each particle is adjusted according to the position update formula. Thirdly, check whether the iteration stopping condition is met. If the global optimal solution is less than the specified error, or if the maximum number of iterations is reached, the iteration is stopped. The optimal weight and threshold of the BP-ANN are output. Finally, this optimal weight and threshold are fed into the BP-ANN, and the BP-ANN is trained until the prediction error is within the set error range or the maximum number of trainings is reached.

In addition, the hidden layer chosen for the PSO-BP integrated model was one, the number of nodes in the input layer was three, the number of nodes in the hidden layer was seven, and the number of nodes in the output layer was one. The training and testing datasets chosen were consistent with the BP-ANN. Other parameters were shown in the Table 5.

## 4. Comparisons of the Semi-Physical Model with Improved Arrhenius-Type, BP-ANN and PSO-BP Integrated Model

### 4.1. Comparisons of the Generative Ability of the BP-ANN and PSO-BP Integrated Model

In order to further estimate the study abilities of these prediction models, the correlation coefficient (*R*) of other evaluation indexes such as Equation (20) was used to estimate the correlation between experimental flow stresses and predict flow stress. A larger value of *R* indicates a good correlation between the two variables, and vice versa.

(20) $$R=\frac{\sum {}_{i=1}^{N}\left(E_{i}-\bar{E}\right)\left(P_{i}-\bar{P}\right)}{\sqrt{\sum {}_{i=1}^{N}{\left(E_{i}-\bar{E}\right)}^{2}{\sum {}_{i=1}^{N}\left(P_{i}-\bar{P}\right)}^{2}}}$$

where *E* is the sample of experimental stress-strain data; *P* is the sample of predicted stress-strain data; *N* is the number of samples of testing dataset.

The *R*-values between the training samples and fitted values of the BP-ANN and PSO-BP integrated model were listed in Table 6. It can be obtained that the *R*-values between the training datasets and fitted values of the BP-ANN and PSO-BP integrated model at different strain rates are larger than 0.999. It can indicate that both the BP-ANN and PSO-BP integrated model can sufficiently and accurately learn the flow behaviors of SAE 5137H steel.

### 4.2. Comparisons of the Predictive Ability of the Three Models

**Figure 10:**
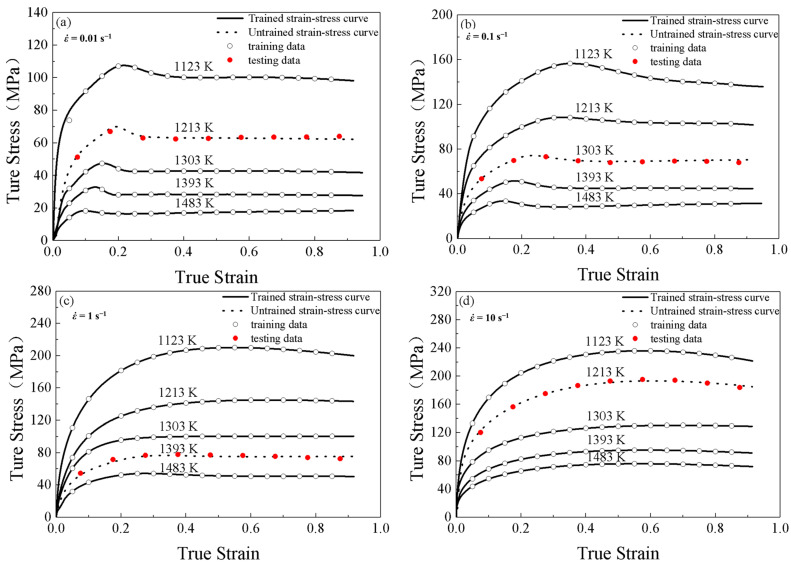
Comparisons between the trained flow stresses and tested flow stresses predicted by the PSO-BP integrated model at different strain rates and temperatures of (**a**) 0.01 s^−1^, (**b**) 0.1 s^−1^, (**c**) 1 s^−1^, and (**d**) 10 s^−1^.

Figure 10 shows the comparisons between stress-strain values predicted by the PSO-BP integrated model and the stress-strain curves obtained by tests at different temperatures and strain rates. It can be seen that the PSO-BP integrated model can accurately track the flow behaviors of SAE 5137H steel under a wide range of temperatures and strain rates.

To further evaluate the research ability of these predictive models, relative error (δ) was introduced as Equation (21).

(21) $$\delta \left(\%\right)=\frac{E_{i}-P_{i}}{E_{i}}\times 100\%$$

where *E* is the sample of experimental stress-strain values; *P* is the sample of predicted stress-strain values.

Another evaluation index average is absolute relative error (*AARE*) as shown in Equation (22), which is the average of the absolute values of *δ*-values. The values of *AARE* were used to further evaluate the research ability of these prediction models. Compared with *δ*-value, *AARE* can better reflect the total prediction error.

(22) $$AARE=\frac{1}{N}\sum {}_{i=1}^{N}\left|\frac{E_{i}-P_{i}}{E_{i}}\right|$$

where E is the sample of experimental stress-strain values; P is the sample of predicted stress-strain values; *N* is equal to the number of samples.

It is worth noting that a larger fluctuation range of δ-values does not mean a worse prediction, and the distribution and relative frequency of δ-values need to be further analyzed using Gaussian distribution. After Gaussian distribution analysis, the mean value of all relative errors μ expressed in Equation (23) can be obtained. The standard deviation (w) expressed in Equation (24) is introduced as an evaluation index to measure the individual dispersion in the dataset. The distribution of relative errors (δ) was measured. Here, a small w indicates that most of the δ-values are close to the μ-value and vice versa. The smaller the μ-value, the closer the predicted stress data are to the experimental stress data.

(23) $$\mu =\frac{1}{N}\sum {}_{i=1}^{N}\delta_{i}$$

(24) $$w=\sqrt{\frac{1}{N-1}{\sum {}_{i=1}^{N}\left(\delta_{i}-\mu \right)}^{2}}$$

where δ is the sample of relative error; μ is the average number of δ-values; *N* is the number of samples of the testing dataset.

**Figure 11:**
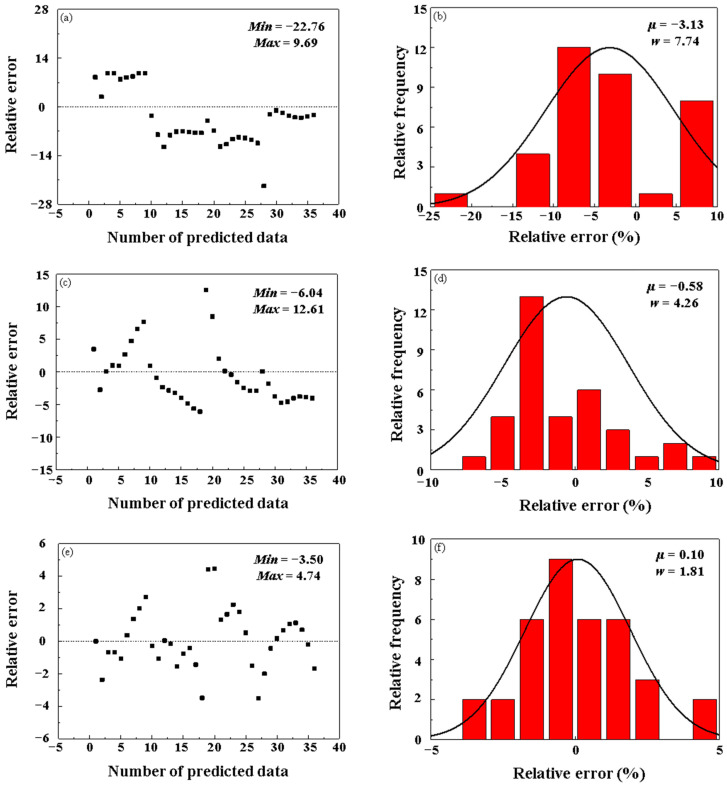
Distributions of the relative errors of the testing data by (**a**,**b**) the semi-physical model with improved Arrhenius-Type, (**c**,**d**) BP-ANN, and (**e**,**f**) PSO-BP integrated model.

Figure 11 shows the δ-value scatter diagrams and histograms of the semi-physical model with improved Arrhenius-Type, BP-ANN, and PSO-BP integrated model, respectively, indicating the relative frequencies of each δ-level. From Figure 10, it can be found that the δ-values acquired from the semi-physical model with improved Arrhenius-Type, BP-ANN, and PSO-BP integrated model vary from −22.76%~9.69%, −6.04%~12.61%, and −3.50%~4.47%, respectively. The μ-value and the w-value of the semi-physical model with improved Arrhenius-Type, BP-ANN, and PSO-BP integrated model are −3.13 and 7.74, −0.58 and 4.26, and 0.10 and 1.81, respectively. In conclusion, the generation ability of the semi-physical model with improved Arrhenius-Type is the worst, while the generation abilities of the BP-ANN and PSO-BP integrated model are at a higher level.

**Figure 12:**
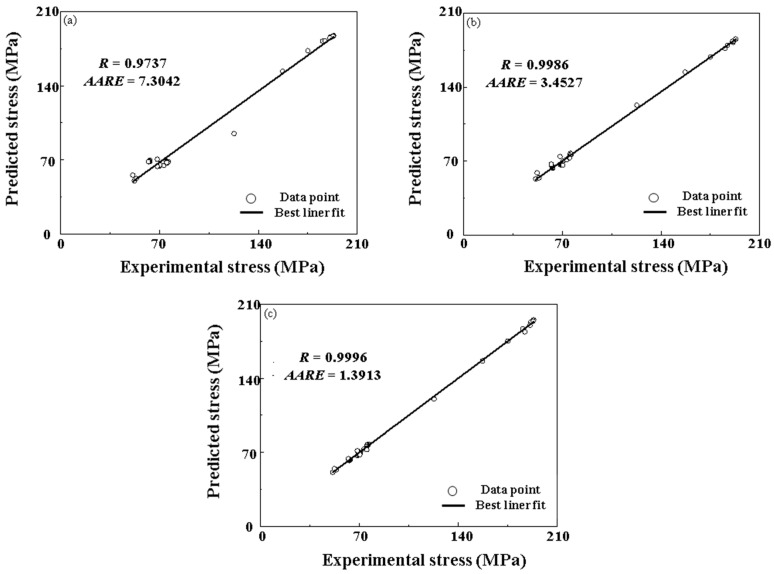
The correlation relationships among the predicted and experimental true stress for the (**a**) the semi-physical model with improved Arrhenius-Type, (**b**) BP-ANN, and (**c**) PSO-BP integrated model.

Figure 12 shows the *R*-values and *AARE*-values of the semi-physical model with improved Arrhenius-Type, BP-ANN, and PSO-BP integrated model test datasets for further comparisons of the generalization ability of these models. The *R*-values and *AARE*-values of the semi-physical model with improved Arrhenius-Type, BP-ANN and PSO-BP integrated model are 0.9737 and 7.3042, 0.9986 and 3.4527, and 0.9996 and 1.3913, respectively. It can be summarized that the PSO-BP integrated model has a larger *R*-value and lower *AARE*-value, which indicates that the PSO-BP integrated model can accurately predict the highly non-linear flow behaviors of SAE 5137H steel. The generation ability of the PSO-BP integrated model is the best, BP-ANN is the second, and semi-physical model with improved Arrhenius-Type is the lowest. The semi-physical model with improved Arrhenius-Type tracks the flow behaviors under a wide range of temperatures and strain rates with the largest error. Worse still, the complex computational procedures of the semi-physical model with improved Arrhenius-Type needs to be recalculated when it comes to some new experimental stress-strain data.

### 4.3. Comparisons of the Modeling Efficiency of the Three Models under Limited Experimental Conditions

Table 7 shows the time in modelling an accurate model of the semi-physical model with improved Arrhenius-Type, BP-ANN, and PSO-BP integrated model. The semi-physical model with improved Arrhenius-Type requires the calculation of a large number of material constants. A large number of multivariate nonlinear regression models were then constructed based on limited experimental data. These material constants and regression models need to be recalculated when new stress data are added. These processes are both complex and time-consuming. In contrast, intelligent algorithms do not need to build complex function models.

The BP-ANN needs to try a large number of network topologies and training parameters to obtain an accurate model, which will consume a lot of time and effort. In addition, the BP-ANN is not very stable. For a certain dataset, the accuracy obtained from different attempts of the same network topology and neural network training parameters fluctuates, which reduces the modeling efficiency. The PSO algorithm has the advantages of few parameters, fast convergence, and strong global search ability. Combining the BP-ANN and PSO algorithms can improve the efficiency and modeling performance of neural network modeling. Therefore, the PSO-BP integrated model has the highest modeling efficiency, BP-ANN has the second, and the semi-physical model with improved Arrhenius-Type has the lowest.

## 5. Applications of the PSO-BP Integrated Model in Material Computations

### 5.1. Stress-Strain Data Expansion by the PSO-BP Integrated Model

**Figure 13:**
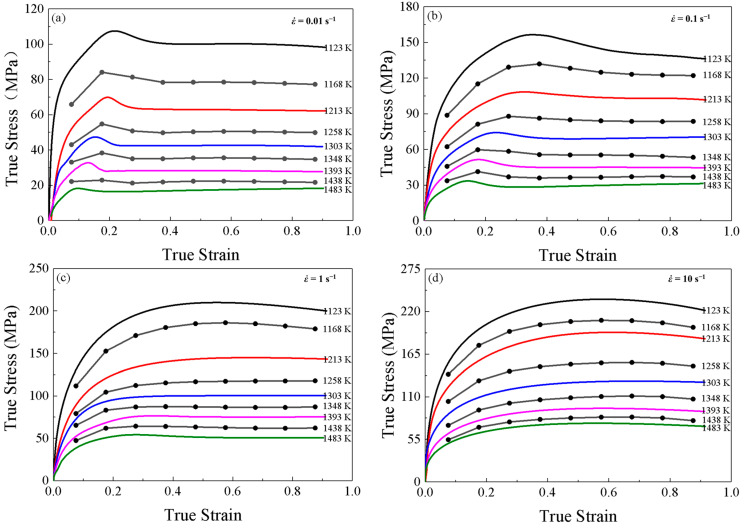
The experimental and predicted results of true stress-strain curves for SAE 5137H steel under different temperatures and strain rates of (**a**) 0.01 s^−1^, (**b**) 0.1 s^−1^, (**c**) 1 s^−1^ and (**d**) 10 s^−1^.

The flow stress data of SAE 5137H steel were predicted using PSO-BP integrated model at temperatures of 1168 K, 1258 K, 1348 K, and 1438 K, and at strain rates of 0.01 s^−1^, 0.1 s^−1^, 1 s^−1^, and 10 s^−1^, and the results were shown in Figure 13. In Figure 13, the solid curves were experimental data and the fitted curves by points were predicted data.

### 5.2. Accuracy Improvement in Finite Element Modeling

If the finite element software needs to invoke stress-strain data that are not initially entered, the software mainly uses mathematical interpolation to calculate the unknown stress-strain data. However, the flow behaviors of materials under different conditions (e.g., different temperatures and strain rates) are complex. The interpolation method cannot predict the stress-strain variation law correctly, resulting in inaccurate simulation results. In this section, the PSO-BP integrated model was used to enrich the stress-strain data, and imported them into the finite element software to simulate the isothermal compression process. A comparison was made with an isothermal compression simulation process that lacks stress-strain data and can only use the FEM’s own interpolation. The experimentally measured stress-strain data were used to simulate the isothermal compression process as a control group.

**Figure 14:**
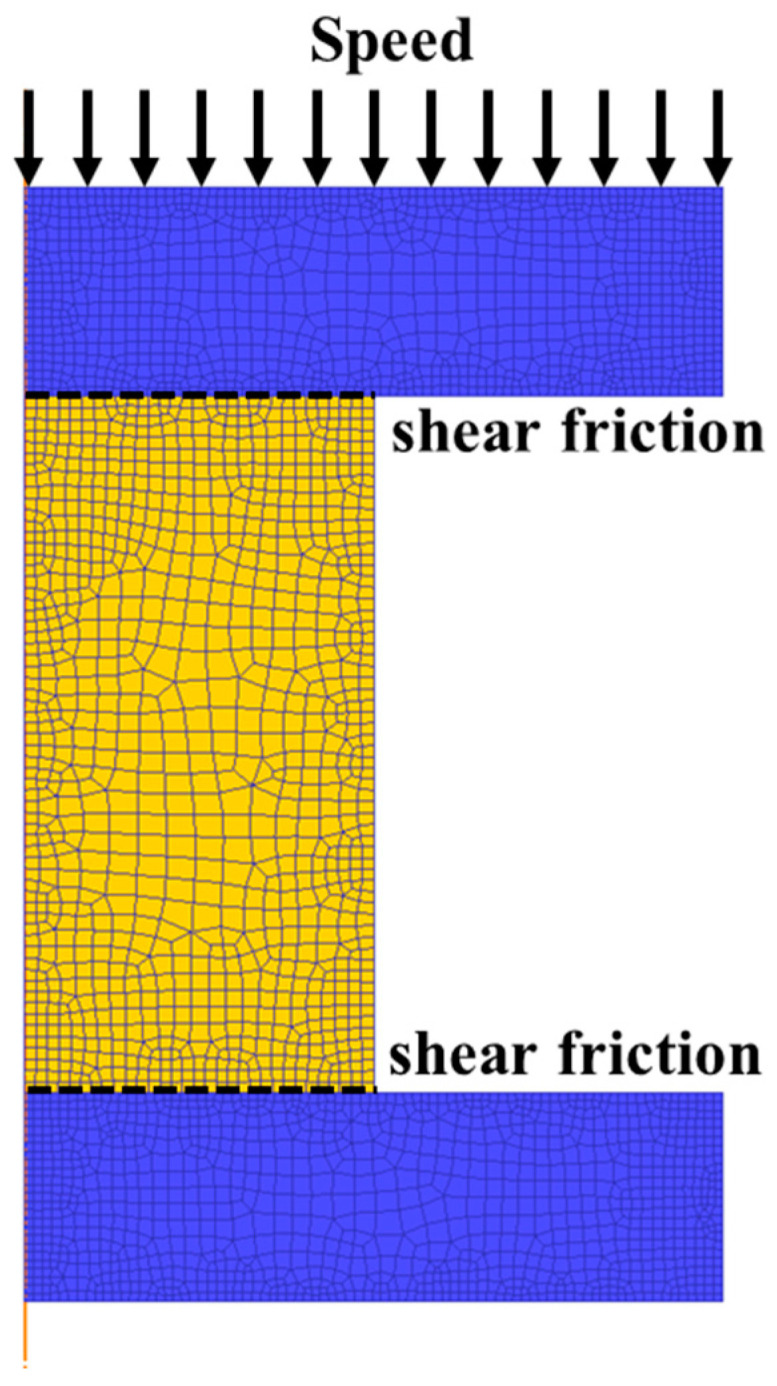
Finite element model for isothermal compression test.

In this section, the effect of the input stress-strain curves on the simulation results of isothermal compression experiments were analyzed using the finite element software, DEFORM. The simulation parameters were set according to the actual experiments. Considering the geometric symmetry of the specimens, half of the specimens were simulated to reduce the computational time. In the actual experiment, the top and bottom surfaces of the specimen were coated with graphite lubricant to reduce the friction between the specimen and the anvil, so the friction type of the contact surface between the specimen and the mold was set to shear type in DEFORM. In addition, the shear friction coefficient is set to 0.3 to simulate the actual graphite lubrication state between the specimen and the anvil. In the finite element simulation, the heat conduction and heat radiation between the compressed specimen, the mold, and the environment were neglected to simulate the experimental isothermal compression test. The finite element model for isothermal compression test is shown in Figure 14. If the finite element software needs to invoke stress-strain data that are not initially entered, the software mainly uses interpolation to calculate the unknown stress-strain data. However, the flow behaviors of materials under different conditions (e.g., different temperatures and strain rates) is complex. The interpolation method does not accurately predict the stress-strain data, resulting in inaccurate simulation results as well. Therefore, the extended stress-strain curves predicted by PSO-BP integrated model were applied to enrich the stress data of SAE 5137H steel.

Three simulation schemes were given in Table 8 for analyzing the effect of the input stress-strain curves on the final simulation results. The initial conditions were identical throughout except for the different input stress-strain curves. Simulated compression tests were performed at a temperature of 1303 K and a strain rate of 0.1 s^−1^. The stress-strain curve at a temperature of 1303 K and a strain rate of 0.1 s^−1^ was entered into the finite element software for scheme-A. Scheme-A has no interpolation interval. Scheme-B used the stress-strain curves predicted by the PSO-BP integrated model. The true stress-strain curves for a strain rate of 0.1 and strain temperatures of 1123 K and 1483 K were input into the PSO-BP model to predict the stress-strain curve for a temperature of 1303 K and a strain rate of 0.1. The data were imported into the finite element software in table format for isothermal compression simulation. Scheme-C used test stress-strain curves with temperatures of a strain rate of 0.1 s^−1^ and 1123 K and 1483 K, so the stress-strain curves with temperatures of 1303 K and strain rates of 0.1 s^−1^ need to be interpolated with an interval of 180 K.

**Figure 15:**
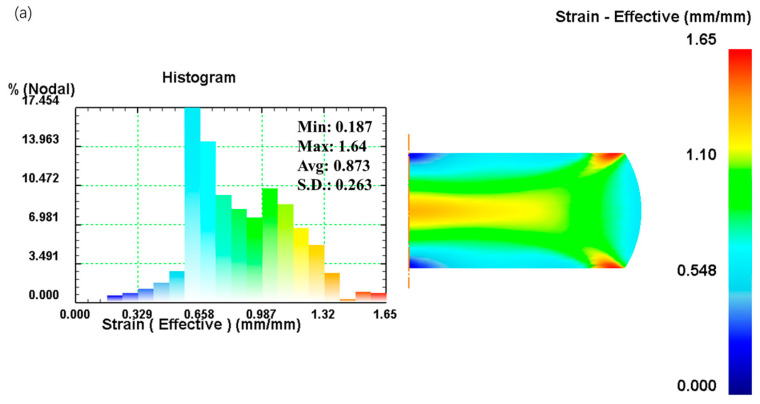

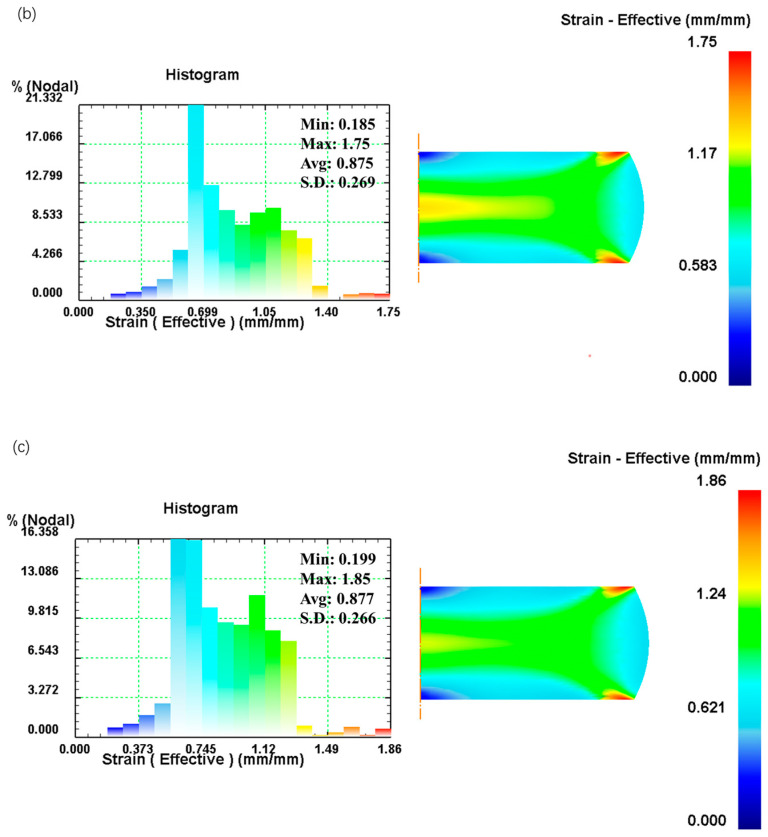
Distributions of effective strain for (**a**) scheme-A, (**b**) scheme-B, and (**c**) scheme-C, at the strain rate of 0.1 s^−1^, the temperature of 1303 K, and the height reduction of 60%.

Figure 15 shows the effective strain distribution of scheme-A, scheme-B, and scheme-C. The effective strain distribution of scheme-B can be roughly divided into five regions. The effective strain distribution of scheme-B is similar to that of scheme-A. The largest strain of scheme-B is 1.75, which is close to that of scheme-A. Figure 13c shows the effective strain distribution of scheme-C, which can also be divided into five regions, but the fifth region is not obvious, and there is a big difference with the effective strain distribution and the maximum effective strain of scheme-A.

**Figure 16:**
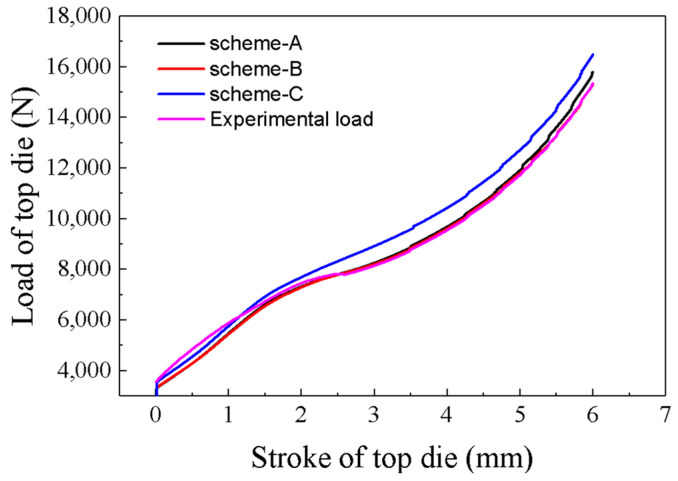
The relationship between the stroke and the loads of top die for these three schemes and experimental values.

In addition, as shown in Figure 16, the load curves corresponding to the upper die stroke for each scenario show that the upper die load curve for scheme-B is very close to that of scheme-A. However, the difference in top die load between scheme-C and scheme-A is large. The relative error of top die load between scheme-A and scheme-B ranged from −0.7090%~3.3506%, and the relative error of top die load between scheme-A and scheme-C ranged from −8.1880%~−4.1680%. Both Scheme-A and scheme-B are closer to the experimentally measured force versus die displacement curves, while scheme-C has a larger gap. It also can be seen from Figure 16 that too large an interpolation span or insufficient stress-strain data can lead to inaccurate simulation results. It shows that PSO-BP integrated model can enrich the flow stress data, reduce the interpolation interval, and improve the simulation accuracy.

## 6. Conclusions

The flow behaviors of SAE 5137H steel were characterized based on the isothermal compression tests at the temperature range of 1123~1483 K and strain rate range of 0.01~10 s^−1^. To accurately and efficiently characterize the complex flow behaviors of this steel, three models including the semi-physical model with improved Arrhenius-Type, BP-ANN, and PSO-BP integrated model were developed according to the obtained stress-strain data. The following main conclusions were acquired from the current study.

(1) Based on experimental stress-strain data, a semi-physical model with improved Arrhenius-Type was developed for the flow behaviors of SAE 5137H steel. A BP-ANN was constructed with temperatures, strains, and strain rates as inputs and stresses as outputs. And a PSO algorithm combined with BP-ANN, namely PSO-BP integrated model also established using the same datasets as BP-ANN.
(2) The comparison results of generative ability between BP-ANN and PSO-BP integrated model show that the correlation coefficient *R*-values were calculated as 0.99997 and 0.99993, respectively. It suggests that the BP-ANN and PSO-BP integrated model has the same excellent generative ability.
(3) The statistical indexes of relative error (*δ*), mean value (*μ*), and standard deviation (*w*) were employed to contrast the predictive ability among the semi-physical model with improved Arrhenius-Type, BP-ANN, and PSO-BP integrated model. The μ-value and w-value for the three models were evaluated as −3.13 and 7.74, −0.58 and 4.26, and 0.10 and 1.81, respectively. It indicates that the predictive ability of PSO-BP integrated model is the best.
(4) The true stress data within the temperature range of 1168~1438 K and strain rate range of 0.01~10 s^−1^ were predicted using PSO-BP integrated model. According to these abundant data, isothermal compression simulation was performed. The results show that the enrichment of stress-strain curves by PSO-BP integrated model is much closer to the reality than the interpolation by FEM software, indicating that PSO-BP integrated model is of great significance for practical applications.

## Figures and Tables

**Table 1:** Chemical compositions of SAE 5137H steel.

C	Mn	Si	S	P	Cr	Mo	Ni	Fe
0.38	1.19	0.28	0.025	0.015	1.23	0.042	0.11	balance

**Table 2:** Polynomial fitting results of $Q\left(\varepsilon \right)$, $n\left(\varepsilon \right)$, $A\left(\varepsilon \right)$ and $\alpha \left(\varepsilon \right)$ of SAE 5137H steel.

$Q\left(\varepsilon \right)$		$n\left(\varepsilon \right)$		$\ln A\left(\varepsilon \right)$		$\alpha \left(\varepsilon \right)$	
B_0_	206.13	C_0_	5.69	D_0_	23.37	E_0_	0.01
B_1_	5471.24	C_1_	25.96	D_1_	371.64	E_1_	0.06
B_2_	−46,988.25	C_2_	−342.05	D_2_	−3350.92	E_2_	−0.65
B_3_	187,335.81	C_3_	1628.67	D_3_	13,856.98	E_3_	2.49
B_4_	−409,247.82	C_4_	−3978.04	D_4_	−31,239.19	E_4_	−4.80
B_5_	505,185.18	C_5_	5347.26	D_5_	39,734.49	E_5_	4.89
B_6_	−331,121.75	C_6_	−3764.62	D_6_	−26,831.79	E_6_	−2.43
B_7_	89,654.64	C_7_	1085.11	D_7_	7484.26	E_7_	0.43

**Table 3:** Specimen division of SAE 5137H steel.

Temperature/K	Strain Rate/s^−1^			
	0.01	0.1	1	10
1123	Training	Training	Training	Training
1213	Texting	Training	Training	Texting
1303	Training	Texting	Training	Training
1393	Training	Training	Texting	Training
1483	Training	Training	Training	Training

**Table 4:** The parameters of the BP-ANN.

Name of Parameter	Parameter Values
Training times	2000
Minimum performance gradient	10^−20^
Learning rate of training	0.02
Adjustment parameters	0.005
Training error target	0.001

**Table 5:** The parameters of the PSO-BP integrated model.

Name of Parameter	Parameter Values
Learning Factor	2
Number of evolutions	100
Learning rate of training	10
random number	[0,1]
Speed range	[−1,1]

**Table 6:** *R*-values between the training datasets and fitted values of the BP-ANN and PSO-BP integrated model under different strain rates.

Strain Rate/s^−1^	*R*-Value	
	BP-ANN	PSO-BP Integrated Model
0.01	0.99990	0.99977
0.1	0.99999	0.99996
1	0.99999	0.99998
10	0.99999	0.99999
Average	0.99997	0.99993

**Table 7:** The time in modelling an accurate model of the semi-physical model with improved Arrhenius-Type, BP-ANN, and PSO-BP integrated model.

Model	Equation	BP-ANN	PSO-BP
The time in modelling an accurate model	More than 180 min	More than 60 min	About 20 min

**Table 8:** The three finite element simulation schemes at the strain rate of 0.1 s^−1^ and temperature of 1303 K.

Temperature/K	Finite Element Simulation Schemes		
	A	B	C
1123	Experimental curve	Null	Experimental curve
1303	Experimental curve	Predicted curve by the PSO-BP	Interpolation of FEM software
1483	Experimental curve	Null	Experimental curve

## Data Availability

Not applicable.
